# The Developing, Aging Neocortex: How Genetics and Epigenetics Influence Early Developmental Patterning and Age-Related Change

**DOI:** 10.3389/fgene.2012.00212

**Published:** 2012-10-17

**Authors:** Kelly Huffman

**Affiliations:** ^1^Department of Psychology, University of CaliforniaRiverside, CA, USA

**Keywords:** cortical gene expression, intra-neocortical connections, brain anatomy, caloric restriction, aging

## Abstract

A hallmark of mammalian development is the generation of functional subdivisions within the nervous system. In humans, this regionalization creates a complex system that regulates behavior, cognition, memory, and emotion. During development, specification of neocortical tissue that leads to functional sensory and motor regions results from an interplay between cortically intrinsic, molecular processes, such as gene expression, and extrinsic processes regulated by sensory input. Cortical specification in mice occurs pre- and perinatally, when gene expression is robust and various anatomical distinctions are observed alongside an emergence of physiological function. After patterning, gene expression continues to shift and axonal connections mature into an adult form. The function of adult cortical gene expression may be to maintain neocortical subdivisions that were established during early patterning. As some changes in neocortical gene expression have been observed past early development into late adulthood, gene expression may also play a role in the altered neocortical function observed in age-related cognitive decline and brain dysfunction. This review provides a discussion of how neocortical gene expression and specific patterns of neocortical sensori-motor axonal connections develop and change throughout the lifespan of the animal. We posit that a role of neocortical gene expression in neocortex is to regulate plasticity mechanisms that impact critical periods for sensory and motor plasticity in aging. We describe results from several studies in aging brain that detail changes in gene expression that may relate to microstructural changes observed in brain anatomy. We discuss the role of altered glucocorticoid signaling in age-related cognitive and functional decline, as well as how aging in the brain may result from immune system activation. We describe how caloric restriction or reduction of oxidative stress may ameliorate effects of aging on the brain.

## Introduction

The human neocortex is the part of the brain that makes us uniquely different from other non-human mammals. Throughout mammalian evolution, the neocortex is the part of the brain that has increased disproportionately in size and complexity, affording our species enhanced abilities including higher-order cognition and reasoning, language, advanced motor skills, and social-emotional behavior. The precise profile of neocortical function results from a series of complicated developmental processes, wherein genes interact with *in utero* and neonatal environmental factors to pattern the structure into a network of functionally and architectonically distinct sensory, motor, and association areas. Developmental neuroscience has made great strides in furthering our understanding of early cortical patterning, however, much less is known about how this patterning is maintained throughout the lifespan of the animal, and even less is known about how the cortex changes throughout aging and senescence.

Scientists who specifically study the biology of aging are developmental biologists and what we learn from studies of early developmental patterning can be applied to the study of the senescent state. This review described age-related change in brain that spans from embryogenesis to the aging adult, and presents the notion that neurological plasticity mechanisms, regulated by both nature and nurture (genetics and epigenetics) are responsible for changes during both early development and late aging. Thus, aging is presented here as an extension of early development, another development time period that occurs late in the animal’s life, but one that relies on similar molecular mechanisms as early development and, like early development can be impacted significantly by both stochastic and epigenetic events.

This review presents research on the developing and aging neocortex, describing how intra-neocortical connections (INCs), which lay the foundation for proper cortical network function, develop in the prenatal and post-natal period. This report investigates the relationship between INC development and cortical gene expression and describes how aging influences gene expression and hence, cortical function. We propose novel concepts surrounding the relationship between cortical gene expression and critical period plasticity and discuss how age-related changes in cortical organization may potentially impact behavior. We review research on immune system contributions to the aging phenotype as well as caloric restriction (CR) and its unique ability to thwart the aging processes that occur in mammalian brain.

Studying of the aging brain, particularly the aging neocortex, represents a tremendously complex and ambitious task. Several approaches must be taken to begin to understand the mechanisms underlying age-related phenotypes in brain anatomy, physiology, and behavior. This review, then, presents research in genetic contributions to aging across lifespan, including early development, specifically highlighting age-related gene expression in the neocortex, but also describes how stochastic, non-programmed events and experience can alter the aging trajectory. How the neocortex is built, maintained, and changed throughout aging is a fundamental issue in neuroscience that deserves great attention. A focus on ways in which experience and epigenetics can ameliorate some of the negative aspects of brain aging is of paramount importance not only to researchers in the field, but also to humans as a species.

## Theoretical Models of Early Neocortical Patterning

**Figure 1:**
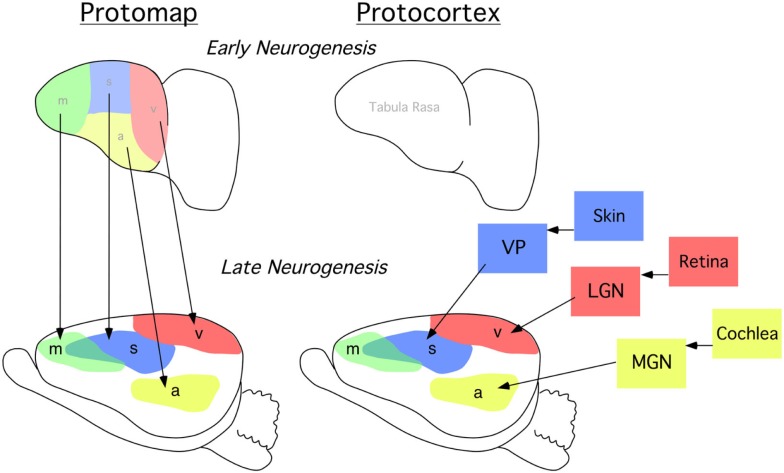
**Classic models of cortical patterning**. Lateral view of cortical primordium at an early stage of neurogenesis (*top row*), and a later stage after thalamocortical axons have begun to enter the cortical plate when putative cortical sensory and motor areas are forming (*bottom row*). (*Left*) simplified protomap model. Early neocortex is patterned by predetermined molecular gradients across the developing cortex. Areal distinctions arise as specified by molecular determinants and these cortically intrinsic factors impart positional areal information. This is an activity-independent process. (*Right*) simplified protocortex model. Early neocortex is considered a blank slate, i.e., “*tabula rasa*.” Area differences arise based on sensory input from axons originating from the dorsal thalamus. Cortical cells and regions are “assigned” sensory and motor territories based on thalamic input. This is an activity-dependent process. m, putative motor cortex; s, putative somatosensory cortex, v, putative visual cortex; a, putative auditory cortex; VP, represents the ventral posterior nucleus of the dorsal thalamus which receives somatosensory input from receptors in the skin via brainstem nuclei; LGN, represents the lateral geniculate nucleus of the dorsal thalamus which receives visual input from the retina; MGN, represents the medial geniculate nucleus which receives auditory input from the cochlea.

All mammalian behavior is generated and regulated by the nervous system. In humans, neocortex is responsible for complex integration of information, the ability to utilize language, decision-making, motivation, and other high-level emotive-cognitive processes and behaviors. The complexity of neocortex emerges during development through a process called arealization, when specific sensory and motor functional areas are formed and connected to one another and to sub-cortical nuclei through a vast and complex network of intra- and extra-neocortical connections. Research on the developmental mechanisms that drive arealization has been influenced by two alternative hypotheses. Rakic (1988) famously detailed his Protomap hypothesis, suggesting that the fate of different neocortical regions were pre-specified in early development by yet-to-be characterized molecules within the proliferative zone, independent of input from the sensory systems (Figure 1, left). The notion that developing neocortex is patterned early in development, regardless of driven sensory input, with differential expression of genes during arealization is highly supported (Rakic, 1988; Miyashita-Lin et al., 1999; Nakagawa et al., 1999; Rubenstein et al., 1999; Bishop et al., 2000; Liu et al., 2000; Ragsdale and Grove, 2001; Zhou et al., 2001; Cecchi, 2002; Nakagawa and O’Leary, 2003; Funatsu et al., 2004; Sansom et al., 2005; Mallamaci and Stoykova, 2006; O’Leary and Sahara, 2008; Rakic et al., 2009; Bedogni et al., 2010). The alternate model, coined the Protocortex Hypothesis, emphasized the role of neural activity, via neocortically extrinsic thalamic sensory input, in determining neocortical areal fate (O’Leary, 1989; Figure 1, right). Based on our experimental finding in the neocortex of a blind mouse bilaterally enucleated at birth, we posit that both cortically intrinsic mechanisms, such as gene expression, and extrinsic mechanisms that involve input from the sensory organs via the dorsal thalamus interact to form the cortical map (Dye et al., 2012).

## Gene Expression and Early Neocortical Patterning

Consistent with the general idea Rakic first proposed, recent results have shown that the developing neocortex is “patterned” early in development, with differential expression of genes during arealization (Donoghue and Rakic, 1999; Miyashita-Lin et al., 1999; Nakagawa et al., 1999; Bishop et al., 2000; Liu et al., 2000; Zhou et al., 2001; Fukuchi-Shimogori and Grove, 2003; Yun et al., 2003; Abu-Khalil et al., 2004; Funatsu et al., 2004; Hamasaki et al., 2004; Shimogori et al., 2004; Sansom et al., 2005; for review see Rubenstein et al., 1999; Ragsdale and Grove, 2001; Ruiz i Altaba et al., 2001; Cecchi, 2002). This patterning is thought to occur independently of sensory input reaching the cortex via thalamocortical afferents, as cortical gene expression patterns are unperturbed in mutant mice lacking these thalamocortical inputs (Miyashita-Lin et al., 1999; Nakagawa et al., 1999). It has been postulated that patterning centers in the midline of the developing telencephalon have a primary role in regulating neocortical regionalization (Rubenstein et al., 1999; Crossley et al., 2001; Fukuchi-Shimogori and Grove, 2001, 2003; Huffman et al., 2004; Sansom et al., 2005). For example, a dorsal patterning center expresses high levels of Bmp and Wnt genes. Mutations that affect Wnt signaling lead to defects in the most medial cortical regions (e.g., the hippocampal complex; Grove et al., 1998; Lee et al., 2000b; Shimogori et al., 2004). Mutations affecting BMP-signaling lead to dorsal-midline patterning defects (Furuta et al., 1997). Additionally, the rostrodorsal midline of the telencephalon expresses high levels of Fgf8; this region is derived from the anterior neural ridge and is known as the commissural plate. Fgf8 has been postulated to regulate aspects of rostral patterning of the telencephalon and its constituents, including the cerebral cortex (Rubenstein et al., 1999).

**Figure 2:**
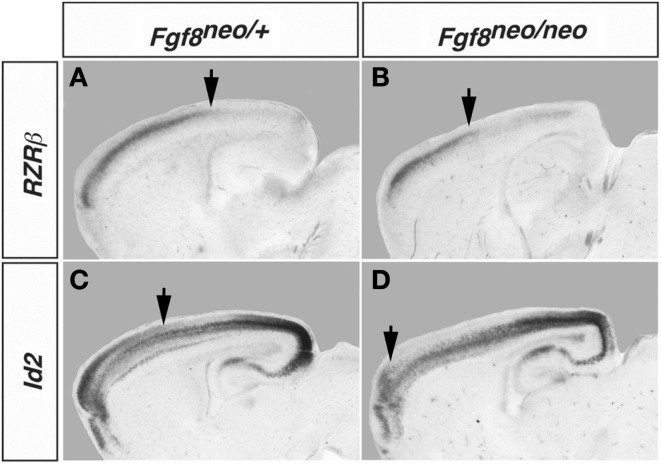
**Shift in *RZR*β and *Id2* expression in the Fgf8^neo/neo^ mutant cortex**. Matched sagittal sections from *in situ* hybridization experiments in control Fgf8^/neo^ **(A,C)** and Fgf8^neo/neo^ **(B,D)** E18.5 mouse brain are shown. There is a rostral shift in the caudal boundary of high *RZR*β expression in the mutant and a rostral shift in the caudal boundary of the superficial domain of *Id2* expression (arrows). Figure adapted from Huffman et al. (2004).

**Figure 3:**
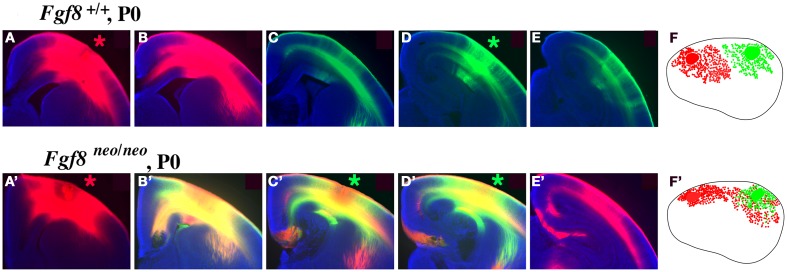
**Intra-neocortical projection patterns in P0 mice with rostral patterning defects (Fgf8^neo/neo^ mutants) compared with P0 control littermates (Fgf8^+/+^)**. Hundred micrometer coronal sections presented in rostral to caudal series of brain hemispheres following DiI [red asterisk, **(A,A′)**] or DiA [green asterisk, **(D,D′)**] crystal placement the rostral and caudal neocortex (putative somatosensory and visual cortex, respectively), oriented with dorsal up and lateral to the right. Sections were analyzed for the distributions of retrogradely labeled cell bodies, with lateral view reconstructions shown in **(F,F′)**. Hemi-sections from control mice **(A–E)** demonstrate no overlap of retrograde label from dye placements in putative somatosensory **(A)** or visual **(D)** cortex, as red and green label remain segregated. However, Fgf8^neo/neo^ mutants showed a robust phenotype, indicated by red–green overlap [yellow label, **(B′–D′)**] and red label caudal to this overlap **(E′)** reflecting ectopic caudal projections to rostral somatosensory cortical locations. The ectopic intra-neocortical connections are easily observed in the reconstructions where caudal locations aberrantly project to rostral fields in the mutant **(F′)** but not in the control **(F)**. **(F,F′)**-Rostral is left, dorsal up. Figure adapted from Huffman et al. (2004).

Mice with altered FGF function in the brain have disrupted cortical arealization, further supporting the Protomap hypothesis (Fukuchi-Shimogori and Grove, 2003; Garel et al., 2003; Huffman et al., 2004; Cholfin and Rubenstein, 2008, Iwata and Hevner, 2009). Cortical gene expression patterns are disrupted in mutant mice with reduced FGF8 signaling. Specifically, a reduction in *Fgf8* expression at the rostral pole of the neocortex leads to a rostral shift of both *RZR*β and *Id2* expression (Figure 2, arrows). This disruption in normal genetic patterning is correlated with ectopic ipsilateral sensory INCs in the mutant (Figure 3). Caudal neurons send projections to far rostral locations, perhaps following the shift in gene gradients (Figures 2 and 3; Garel et al., 2003; Huffman et al., 2004). Results from these studies and others have demonstrated that Fgf8 plays a regulatory role in the development of intra-neocortical connectivity and led our laboratory to further investigate gene expression-INC relationships (Fukuchi-Shimogori and Grove, 2001, 2003; Garel et al., 2003; Huffman et al., 2004; Shimogori and Grove, 2005; Dye et al., 2011a,b). We have examined the gene expression patterns of seven regulatory genes that are expressed in specific regions or gradients across the cortical sheet in early development (Miyashita-Lin et al., 1999; Garel et al., 2003; Huffman et al., 2004; Sur and Rubenstein, 2005) from the embryonic period to adulthood in mouse and studied their relationship to INC development. These genes, which are showcased in two recent reports (Dye et al., 2011a,b), are believed to be involved in the process of area and areal boundary formation as expression patterns often correlate with emergence of area borders in development (Dye et al., 2011a). The seven genes included in the analyses were *COUP-TFI*, *Id2*, *RZR*β, *Cadherin 8*, *Ephrin A5*, *Eph A7*, and *Lhx2* (Dye et al., 2011a,b), some of which are shown in this review.

## Cortical Areas and Their Connections: INCs and Gene Expression from Embryogenesis through Adulthood

**Figure 4:**
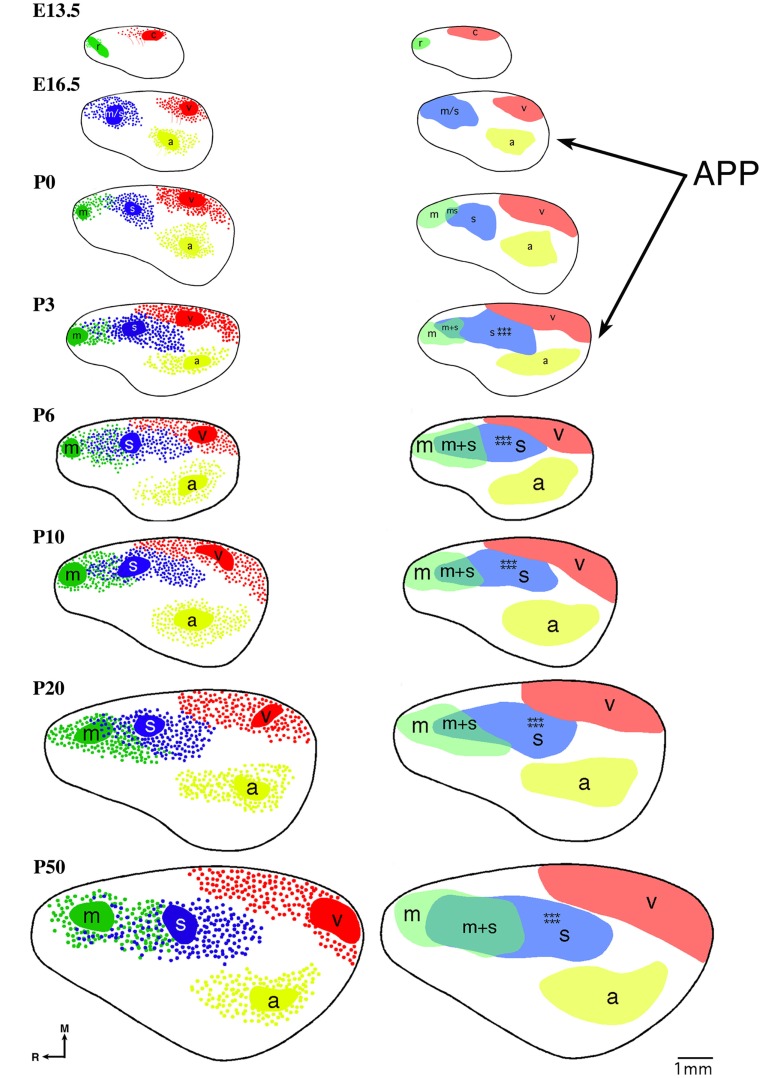
**Reconstruction of areal boundaries through analysis of intra-neocortical connections in E13.5-P50 wild-type mice**. All panels represent a lateral view of one hemisphere. Left column: dye placement locations and organization of retrogradely labeled cells (colored patches, dye placement, and dye spread; red filled circles, retrogradely labeled cells in putative caudal/visual cortex; blue filled circles: retrogradely labeled cells in putative somatosensory cortex; green filled circles, retrogradely labeled cells in putative rostral/motor cortex; yellow filled circles, retrogradely labeled cells in putative auditory cortex; thick black line, hemisphere outline). Right column: lateral view reconstructions of putative areal boundaries as determined by INC analyses (colored areas, putative cortical areas as labeled; r, putative rostral area; c, putative caudal area; m, putative motor cortex; m + s, putative sensory-motor amalgam; m/s or s, putative motor/somatosensory or somatosensory cortex; a, putative auditory cortex; v, putative visual cortex). Areal patterning period (APP) is from E16.5-P3. Stars indicate location of putative barrel field. Oriented medial (M) up and rostral (R) to the left. Scale bar = 1 mm. Adapted from Dye et al. (2011a,b).

**Figure 5:**
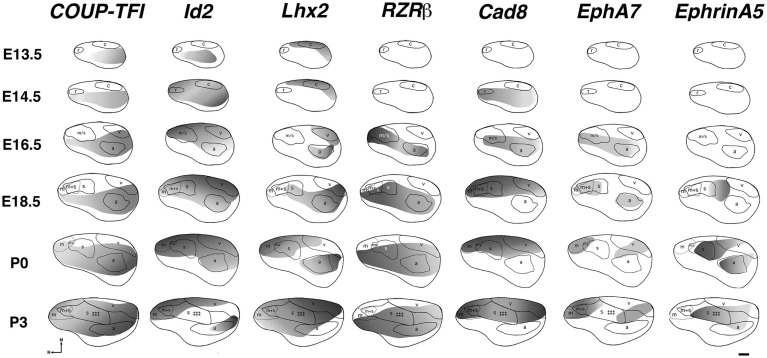
**Lateral view reconstructions of *COUP-TFI*, *Id2*, *Lhx2*, *RZR*β, *Cad8*, *EphA7*, and *EphrinA5* gene expression gradients or gene maps (gray shaded areas) coregistered with areal reconstructions of E13.5-P3 brain hemispheres**. R, putative rostral area; c, putative caudal area; m, putative motor cortex; m + s, m/s, putative sensory-motor amalgam; s, putative somatosensory cortex; a, putative auditory cortex; v, putative visual cortex. Stars indicate location of barrel field. Oriented medial (M) up and rostral (R) to the left. Scale bar = 500 μm. Adapted from Dye et al. (2011a).

**Figure 6:**
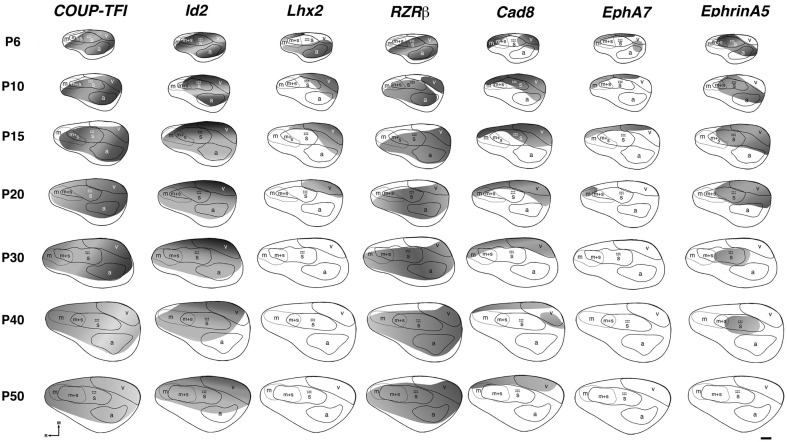
**Lateral view reconstructions of *COUP-TFI*, *Id2*, *Lhx2*, *RZR*β, *Cad8*, *EphA7*, and *EphrinA5* gene expression gradients or gene maps (gray shaded areas) coregistered with areal reconstructions of P6-P50 brain hemispheres**. R, putative rostral area; c, putative caudal area; m, putative motor cortex; m + s, m/s, putative sensory-motor amalgam; s, putative somatosensory cortex; a, putative auditory cortex; v, putative visual cortex. Stars indicate location of barrel field. Oriented medial (M) up and rostral (R) to the left. Scale bar = 500 μm. Adapted from Dye et al. (2011b).

**Figure 7:**
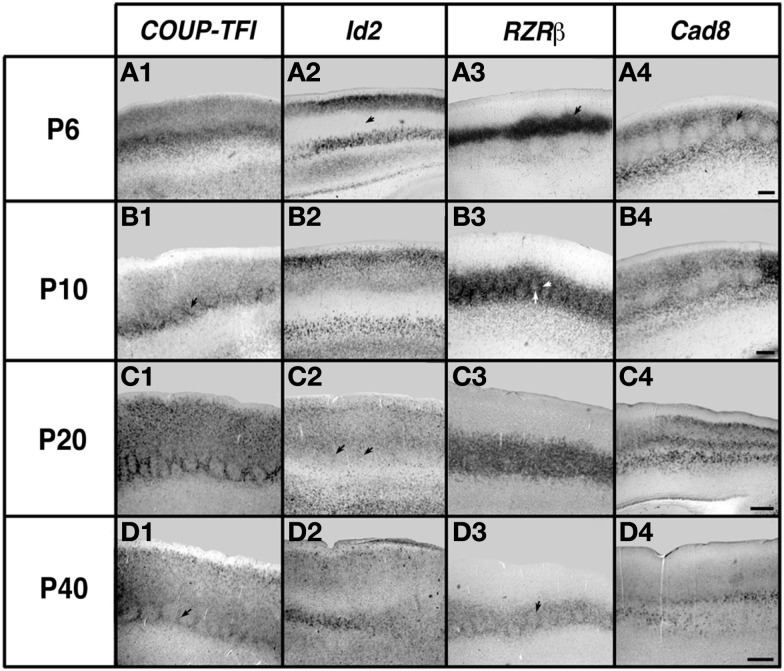
**Analysis of neocortical gene expression in the barrel field of somatosensory cortex**. One hundred micrometers sagittal sections of brain hemispheres after *in situ* RNA hybridization with probes against *COUP-TFI*, *Id2*, *RZRβ*, and *Cad8*. Ages examined for each gene: P6, P10, P20, and P40. Oriented with dorsal up and rostral to the left. *COUP-TFI* exhibits robust expression the barrels at P6, and at later ages expression is mostly restricted to barrel septa (outlines of barrels, arrows in B1, D1). *Id2* expression in the layer 4 barrel field is absent at P6 (arrow in A2), but some light expression in septa can be seen at P20 (arrows in C2). *RZR*β exhibits robust expression in the barrel septa and hollows at P6 (arrow in A3), and although expression continues in both septa and hollows at later ages, the septal expression become relatively stronger (arrows in B3, D3). *Cad8* displays limited expression in the barrel hollows but strong expression is seen in the septa at P6 and P10 (arrow in A4). Specific barrel expression is lost at P20 and P40 (C4, D4). Scale bar = 100 μm. Adapted from Dye et al. (2011b).

The earliest sensory INCs documented in mice were found in the caudal cortex, in a location corresponding to developing visual cortex on embryonic (E) day 13.5 (Dye et al., 2011a, Figure 4). This growth of projections was correlated with *Lhx2* expression and flanked by expression of *Id2* and *COUP-TF1* (Figure 4, top and Figure 5, top row). The areal patterning period (APP), or the time in which features of neocortical sensory and motor areas are established, was described and defined as the period from embryonic day (E) 16.5 to post-natal day (P) 3 in mice (Dye et al., 2011a, Figure 4). This APP occurs before eye opening and active whisking and represents a period of time where the distribution of INCs matures into an adult-like pattern, present as early as P3, where the borders of cortical sensory and motor areas are distinct and similar to what is observed in adults (Figure 4). Interestingly, although INC patterns remain fairly stable from P3 to P50 (early adulthood, Figure 4), gene expression patterns do not (Figures 5 and 6). Specifically, most expression patterns of the seven genes tested (see above list) in Dye et al. (2011a,b) either lose the location specificity of expression as the mouse ages, or decreases significantly over time (Figure 6). For example, *Lhx2* expression correlated with developing visual and auditory cortical areas from E13.5-P20, after which expression becomes undetectable in cortical tissue (Figures 5 and 6).Of the seven genes we tested, four were expressed into adulthood but showed decreased levels of expression (*COUP-TFI*, *Id2*, *Cad8*, and *RZR*β) across the cortex. Also, the expression of these four genes in the barrel field differed dramatically from the onset of barrel field formation to the adult barrel pattern (Figure 7). For example at P40, *COUP-TF1* expression is dense in the barrel septa, where it was not present at P6 (Figure 7). This directed our hypothesis that genes expressed in cortex during adulthood may have switched their functional role from developmental to maintenance of cortical area borders or features.

**Figure 8:**
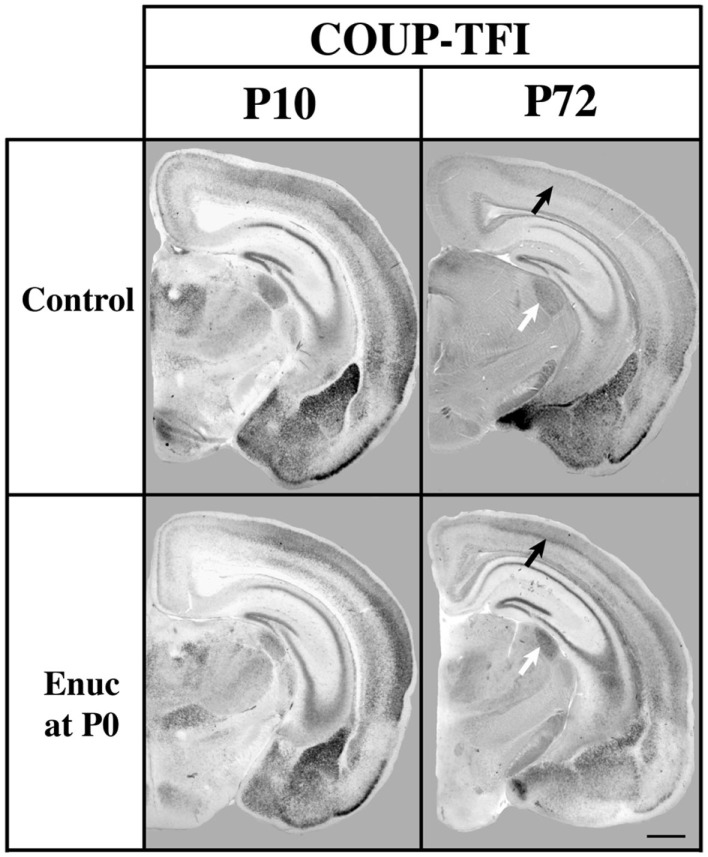
**Analysis of *COUP-TFI* gene expression in P10 and P72 control mice and mice bilaterally enucleated at birth**. One hundred micrometers coronal sections of P10 and P72 brain hemispheres following *in situ* hybridization with a probe against *COUP-TFI*. At P10 strong *COUP-TFI* expression is seen in layer 4 of the caudo/lateral cortex in both control and enucleated animals. Layer 4 *COUP-TFI* expression at P72 is maintained in the enucleated animal, but is notably reduced in control animals (black arrows).Additionally, expression of *COUP-TFI*, although present in a smaller domain due to the decreased sized of the nucleus after enucleation, shows increased expression in the LGN of mice enucleated at birth as compared to controls at P72 (white arrows). Normal developmental time limits of *COUP-TFI* expression in mouse brain are extended by removal of visual activity, perhaps representing an extension of the critical for plasticity. Oriented dorsal up and lateral to the right. (Data from a published abstract, Society for Neuroscience conference, Huffman et al., 2010).

Our study of gene expression and INCs in the *Fgf8* mutant and the normal wild-type mouse spanning from embryogenesis to adulthood led to the idea that gene expression not only regulates INC position but that the decline of cortical gene expression throughout life correlated with period closures of sensory critical periods. If, indeed, gene expression regulates critical period closure, we posit that sensory deprivation, which is known to extend the critical period for plasticity in cortex, would also extend the decline of gene expression. In a P72 mouse bilaterally enucleated at birth, we observed increased expression of *COUP-TFI* present in the caudal neocortex when compared to levels of expression in control mice (Huffman et al., 2010; Figure 8). This extension of normal gene expression in a mouse with long term visual deprivation supports our hypothesis that natural reduction of gene expression in cortex with age plays a role in closure of critical periods for plasticity and that sensory deprivation may extend critical periods via extension of cortical gene expression.

The studies described above in the developing mouse highlight the Protomap model and speak to the importance of gene expression in early neocortical patterning. However, based in our work in an enucleated mouse, where bilateral enucleation at birth not only altered the pattern of the INCs and the neocortical network, but also generated a shift in gene expression (Dye et al., 2012); it becomes clear that the role of epigenetics and experience cannot be ignored. Although epigenetic change is not heritable, the plasticity mechanisms that allow for the change are, and we believe that these genetically mediated plasticity mechanisms, which are poorly understood at this point, serve as a building block for age-related change.

## Neocortical Aging and Gene Expression

We have demonstrated that the precise development of at least one aspect of cortical anatomy, the INCs, is regulated by gene expression (Huffman et al., 2004; Figures 2 and 3). Furthermore, through a comprehensive developmental analysis from embryogenesis through adulthood, we have correlated changing patterns of gene expression in neocortex as changes in functional anatomy emerge and are maintained (Dye et al., 2011a,b; Figures 4–7). These studies only assayed a small number of genes that were previously thought to be involved with the establishment of cortical areas in development, and did not extend into late adulthood. In an attempt to determine molecules or sets of molecules that may be involved in aging of specific tissues at specific locations, several laboratories have used microarray technology to survey great numbers of genes in neocortex and other brain regions of aging and control mice. The first published study on this topic cast a global analysis of age-related changes in mouse brain gene expression using oligonucleotide arrays analyzing 6,347 genes. Researchers found changes in cortical mRNA expression in 63 (about 1%) of the genes studied. Interestingly, 13 of these genes (20%) were related to an immune response in the neocortex (Lee et al., 2000a). Since then, several groups have used microarray in mouse model to look for up- or down-regulation of genes within the aging brain, with several studies showing most changes present in the prefrontal cortex and many correlating with immune system response (Jiang et al., 2001; Prolla, 2002; Zahn et al., 2007; Chen et al., 2010; Kedmi and Orr-Urtreger, 2011). Recently, researchers have found upregulation of microRNAs during aging in the mouse brain and have suggested that microRNA upregulation begins in mid-life and that extreme longevity is correlated with more stability and less upregulation of those microRNAs in later life (Li et al., 2011). Others have found developmental changes in microRNA expression in brain throughout the life of the animal, indicating the potential role of microRNAs in cell proliferation and brain growth, as well as in mechanisms related to brain aging (Eda et al., 2011). This provides some support for the notion that similar mechanism involved in early brain growth and patterning may also be involved in age-related change.

Although the vast majority of genetic studies of the aging brain have been done using microarray technology in the mouse model, some groups have used this and other techniques to study age-related changes in both human and non-human primate brains. Most genetic studies conducted in human brain direct their focus on the prefrontal cortex. Researchers have reported that several genes involved in synaptic and mitochondrial function are downregulated after about age 40 in human prefrontal cortex, which is, in turn, followed by an induction of an immune response not dissimilar to that described in mouse models of brain aging (Lu et al., 2004). DNA damage has been observed in the promoters of genes with reduced age-related expression, and it has been suggested that the promoter damage resulted from oxidative stress in the cells, a theory that has recently become popular among aging researchers (see below; Lu et al., 2004).

Another study in human prefrontal cortex noted microarray gene changes of 540/22,000 genes studied in the aging brain (Erraji-Benchekroun et al., 2005). Most downregulated genes showed neuron-enriched transcripts that are most likely involved in cell–cell communication and circuitry, whereas upregulated genes were potentially linked to the immune response.

Studies in non-human primates and those that included human and animal models have been very informative, providing much needed comparative genetic analyses. Fraser et al. (2005) found that human and monkey brains aged differently. Specifically, the human and chimpanzee cortex showed different age-related changes in molecular profiles, showing great dissimilarity between the two primate species. However, in a study of humans and macaques, a subset of gene changes were found to be conserved between the species including a significant age-related upregulation of the neuroprotective gene apolipoprotein D (APOD) and down-regulation of the synaptic cAMP signaling gene calcium/calmodulin dependent protein kinase IV (CAMK4; Loerch et al., 2008). This change in APOD was also reported as a frequent finding across many studies in a recent meta-analysis of age-related change in gene expression in studies of rodent and human brain (de Magalhães et al., 2009). Although Loerch et al. (2008) demonstrate significant differences between primate and mice species in their analysis of age-related changes in brain gene expression, human and primate comparative microarray data suggest that neuronal genes tend to be downregulated with age; a finding consistent with our findings in the maturing CD-1 mouse where most transcripts reduce in specificity with age (Loerch et al., 2008; Dye et al., 2011b; Figures 5 and 6).

Although changes in gene expression are present in the aging brain, it does not prove that these age-related alterations are from a cell program dictating fate, as is supported in early developmental patterning of brain tissue. There is evidence that stochastic, non-deterministic changes in the post-natal brain may play a significant role in the aging brain. Although changes in global gene expression have been observed, variation among individual cells has also been described (Bahar et al., 2006) which is most likely from genomic instability that produces the non-systematic alteration in gene expression level. Bahar and colleagues have reported cell mutations that accumulate with age at an organ- and tissue-specific rate. They suggest that stochastic genomic instability plays a critical role in the aging process through the impact of stochastic mutations that alter normal gene expression, producing cellular degeneration, and death (Vijg et al., 2005). Given the observed age-related changes in gene expression in the mammalian brain, it is quite plausible that stochastic deregulation of gene expression directly leads to cellular degeneration and death that adversely impacts normal cognitive function (Bahar et al., 2006). The ability for stochastic deregulation of gene expression to occur could quite possible represent a heritable plasticity mechanism that allows for both age-related and evolutionary change in neocortical structure and function. As tends to be true in most nature vs. nurture debates, the most likely explanation is that both age-related changes in genetic program and stochastic changes in genomic stability together influence the aging cellular structure and function.

## Aging and Cortical Neuron Morphology and Wiring

Although our published studies of INC development in the adult mouse, described in a previous section, ended in early adulthood, we have recently analyzed the patterns of INCs of somatosensory, visual, and motor cortex in 18-month-old mice and did not find any phenotypic differences in the overall number of projections from the cortical areas (Abbott et al., 2012). Our data in mouse are consistent with studies in primates that have not shown a decrease in the number of neurons in the hippocampus or neocortex related to aging (Peters et al., 1998; Peters and Rosene, 2003). However, aging does appear to significantly impact myelinated nerve fibers as well as some microstructural changes in neocortical anatomy. Specifically, Peters and colleagues have published several reports on structural changes, such as increased lipofuscin within cells and loss of dendritic spines in the cerebral cortex of rhesus macaque monkeys (Peters et al., 1998; Page et al., 2002; Peters and Rosene, 2003; Peters, 2007, 2009; Peters and Kemper, 2012). The cell type within neocortex that seems to demonstrate the greatest age-related alteration is neuroglia (Peters, 2007). Although some structures such as the anterior commissure (Sandell and Peters, 2003), and layer 1 in cortical areas 17 and 46 (Peters and Sethares, 2002) do not show a significant change in neuroglial numbers with age; there is a 45% age-related increase in the number of oligodendrocytes in area 46 (Peters and Kemper, 2012), a 20% increase in fornix (Sandell and Peters, 2002), and a 50% increase in area 17 (Peters and Kemper, 2012). These age-related changes in glia appear to be area-specific, could be generated from an integration of genetic and epigenetic processes and may be relate to functional changes in neocortex that lead to cognitive decline in aging.

The integrity of the intra-neocortical circuit is critical for proper cognitive function. Natural age-related demyelination is observed in the primate model and may represent damage to the intra-neocortical circuitry, which, in turn, could lead to cognitive decline (Peters, 2009). Peters and Kemper (2012) posit that age-related cognitive decline correlates with and possibly results from problems with myelination, altered intra-neocortical connectivity, and abnormal synaptic and dendritic function.

Patrick Hof’s group has also made significant inroads in understanding age-related anatomical changes in the primate brain. Specifically, they reported that in Alzheimer’s disease, pyramidal cells and their projections were particularly vulnerable to cell death (Morrison and Hof, 2002). In normal aging, although the cells did not appear vulnerable to death, their function was greatly impacted by other anatomical changes such spine loss, and a decrease in NMDA receptor signaling in hippocampal tissue. Thus, they posit that these sub-lethal changes in neurons and their circuits could play a role in normal age-related cognitive decline. In a study of short- and long-range INCs in non-human-primates, Hof’s group found that levels of GluR2 and NMDAR1 glutamate receptor subunit protein immunoreactivity were significantly decreased in a proportion of projection neurons in cortex (Hof et al., 2002). They found that both types of projection neurons showed age-related neurochemical changes. Furthermore, a study in aging Patas monkeys examined the INCs between temporal and prefrontal cortex (Page et al., 2002). In this primate species, projection neurons demonstrated a loss of dendritic spines at all levels of their dendritic trees. In a follow up study, Hof and colleagues describe differences in dendritic spine loss in different positions on the dendrites of projection neurons in macaque cortex (Duan et al., 2003). They found the biggest spine reduction in the proximal portion of apical dendrites, with an overall reduction observed throughout. On the basal dendrites, the greatest reduction of spines was located on the distal branches. Despite an overwhelming amount of data suggesting that neuron loss is not associated with normal aging (Peters et al., 1998; Morrison and Hof, 2002; Peters and Rosene, 2003), the alterations in the dendritic branches of important projection neurons in aging monkey cortex described above suggest that subtle changes in brain anatomy can have significant impact on brain function, and thus, cognition (Duan et al., 2003; for review, see Morrison and Hof, 2007). Recently, researchers from Croatia were able to reverse these age-related effects on projection neuron dendrites through the use of environmental enrichment highlighting the impact of epigenetics on the aging brain. In their study in a rodent model, the age-related loss of dendritic spines on projection neurons was ameliorated in a group of aging rats exposed to an enriched housing environment (Rasin et al., 2011). As in early development, this implicates the role of sensory input in shaping and maintaining structural aspects of the brain.

Although tract tracing data from our laboratory in 18-month-old aging mice show no significant changes in area-to-area global projections in neocortex, it is highly possible that changes in myelin and cortical microstructure including dendritic spine number and morphology are affected in a murine model of aging (Abbott et al., 2012). This warrants further study at the microstructural level.

Studies utilizing standard anatomical techniques in monkeys have been replicated in both humans and primates using diffusion tensor imaging (DTI), also called diffusion tensor magnetic resonance imaging (DT-MRI). For example, DTI studies in aging macaque monkeys showed a loss of myelinated axons within the neocortex without any obvious loss of gray matter thickness. Most significant changes were found in the major fiber tracts of the frontal lobe (within the anterior corpus callosum) where myelination was reduced (Makris et al., 2007). These findings corroborate theories implicating the role of frontal lobe function in age-related human cognitive decline. Although these findings suggest a greater phenotype in more rostral regions, other studies have shown more global effects of age-related changes in myelination (Moy et al., 2011). For example, DTI analyses revealed white-matter axonal changes not only in frontal areas but in multiple regions throughout neocortex as well. These white-matter changes were related specifically to impaired reaction time in a simple reaction time task (Moy et al., 2011). Another human DTI study demonstrated microstructural changes in the white matter of multiple cortical regions in older people diagnosed with mild cognitive impairment (Cho et al., 2008).

The neocortex is a living, complex structure that continues to change molecularly, structurally, and physiologically throughout the lifespan of the animal. The structural components of the brain are first patterned by interplay of genetic and epigenetic influences, are maintained through both gene expression and neural activity, then continue to develop and change later in life. The degradation of myelin structures decreases function and slows processing, which has a profound effect on the intra-neocortical circuitry that is critical for normal cognitive function. Although neuronal cell death does not appear to be a major issue in the non-diseased aging brain (Peters et al., 1998; Morrison and Hof, 2002; Peters and Rosene, 2003), the ability of neurons to function properly is greatly impacted by changes in microstructure, including loss of dendritic spines and myelination. As we have suggested previously that neocortical gene expression plays a role in the development and maintenance of cortical structure, and that patterns of gene expression within neocortex change throughout the life of the animal, it follows that gene expression may be involved in age-related change in the brain, specifically age-related changes that may impact the brain’s structure and neuronal microstructure, and hence, cognitive function. However, given the experience-related effects on brain anatomy and even gene expression, epigenetics must interact with gene expression to create the aging phenotype.

## The Immune System and the Aging Brain

As mentioned previously, some of the observed anatomical change in dendrites or myelinated axons in the aging brain may be related to immune mechanisms. A recent paper has documented some very interesting changes in immune gene regulation in the neocortex of aging mice. Specifically, researchers have correlated age-related impairments in motor behaviors, cognition, and motivation with upregulation of serum cytokine levels in the medial prefrontal cortex (Bordner et al., 2011). The medial prefrontal cortex is a part of the neocortex that has been frequently discussed as a potential key player in mild cognitive impairment observed in aging humans (Allard et al., 2012; Caetano et al., 2012). Finally, glucocorticoids are molecules that have been shown to alter cerebral cortex development in clinical and animal studies of perinatal exposure (Antonow-Schlorke et al., 2003; Mutsaers and Tofighi, 2012; Zuloaga et al., 2012). Glucocorticoids have also been implicated in age-related degradation of brain anatomy and thus age-related decline of cognitive function (Holmes et al., 2010; Soontornniyomkij et al., 2010). Exactly how and to what extent glucocorticoid exposure can impact cortical microstructure throughout the lifespan of the animal is an area that warrants further study.

Another possible immune system related mechanism is related to epigenetic effects, not unlike the impact of stochastic changes in the system that perturb gene expression patterns of individual cells. It has been reported recently that senescent cells secrete factors that impact the surrounding tissue (Rodier et al., 2009). Specifically, DNA damage resulting from DNA double-strand breaks in aging initiates the secretion of inflammatory cytokines such as interleukin-6. This release of cytokine was specifically related to cellular senescence and may significantly impact the cellular phenotype. It is quite plausible that groups of these senescent cells that secrete cytokines from DNA damage could greatly impact patterns of gene expression in aging brain. Subsequent change in gene expression, could thus impact features of brain anatomy, as is observed in early developmental time periods. Neocortical anatomy, which is most often related to cognitive function in humans, may be adversely affected by this stochastic immune response.

## Caloric Restriction and the Aging Brain

After first documenting many age-related changes in multiple tissues including brain gene expression from microarray studies in aging mice, Prolla’s research group has advanced our knowledge of this research area substantially by investigating the impact of CR on age-related changes in gene expression throughout the body. First, in 1999, it was reported that age-related changes (up-or down-regulation) in gene expression in mouse skeletal muscle were partially or completely prevented through a reduction in dietary caloric intake (Lee et al., 1999). This was a landmark study that presented the now popularized and widely accepted notion that CR in mammals retards the aging process. Subsequently, Prolla presented the hypothesis that the prevention of age-related changes in gene expression with CR potentially resulted from the decrease in oxidative stress that occurs during CR. They suggested that CR provides a neuroprotective effect on the degrading, aging brain, particularity the cerebral cortex (Prolla and Mattson, 2001; Weindruch et al., 2001). More recently, several laboratories have demonstrated the robust effect of CR on the attenuation of aging in the brain, particularly demonstrating phenotypic prevention of gene up- or down-regulation effects in measures of mRNA and microRNAs in brain tissue (Khanna et al., 2011). Genes associated with apoptosis in aging are downregulated and the age-related decrease of SIRT1 expression, a longevity factor that appears to play a neuroprotective role in neuropathological disease, in cerebral cortex that has been reported by several laboratories has been shown to be prevented in late-onset CR (Chen et al., 2008; Khanna et al., 2011; Quintas et al., 2012). Age-related increases in 5-hmC, thought to be involved in regulation of gene expression in brain, and age-related changes in Nnmt-3a, a molecule that catalyzes DNA methylation, are prevented by CR (Chouliaras et al., 2011, 2012).

## Conclusion

There is a vast amount of data demonstrating the relationship between gene expression and anatomical features in early brain development, including neocortical circuit development, as well as late-stage changes in gene expression and circuitry in the aging animal. Based on our research that spans the lifespan from the embryonic period to adulthood as well as data from those studying the aging animal model, we suggest that similar genetic and epigenetic mechanisms continue to impact the structure and function of the brain throughout life. Early on, both genetics and experience guide neocortical and brain patterning, and these mechanisms continue to impact the maintenance of cortical areas and their boundaries as well as physiological area function throughout adulthood. Late in life, similar genetic mechanisms may be involved in the breakdown of brain microstructure, and changes in experience, as in early development, can either advance or ameliorate the deleterious effects of aging. Data from multiple laboratories around the world suggest that CR and environmental enrichment can impact gene expression in aging brain, which most likely affects microstructural aspects of cortical architecture. This research represents an exciting direction that will greatly advance our knowledge of the aging process in mammals.

## Conflict of Interest Statement

The author declares that the research was conducted in the absence of any commercial or financial relationships that could be construed as a potential conflict of interest.
